# Biomarkers and Preclinical Models for Adult T-Cell Leukemia-Lymphoma Treatment

**DOI:** 10.3389/fmicb.2019.02109

**Published:** 2019-09-18

**Authors:** Lee Ratner

**Affiliations:** Division of Oncology, School of Medicine, Washington University in St. Louis, St. Louis, MO, United States

**Keywords:** adult T-cell leukemia-lymphoma, preclinical, xenograft, mouse models, tax, HBZ, biomarkers

## Abstract

Adult T-cell leukemia/lymphoma (ATL) is an aggressive lymphoproliferative malignancy with a very poor prognosis. Despite several recent advances, new therapeutic approaches are critical, and this will require successful preclinical studies, including studies in ATL cell culture systems, and mouse models. Identification of accurate, reproducible biomarkers will be a crucial component of preclinical and clinical studies. This review summarizes the current state-of-the-art in each of these fields, and provides recommendations for future approaches. This problem is an important unmet need in HTLV research.

## Introduction

Adult T-cell leukemia-lymphoma (ATL) is a refractory T cell malignancy. The most common clinical variants, acute and lymphoma subtypes, are associated with a median survival of less than 1 year (Katsuya et al., 2015). More indolent variants, smoldering and chronic ATL, are also associated with poor prognoses, with median survivals of 2.6 and 4.6 years, respectively. In the last several years, substantial progress was made in therapy for ATL. These achievements include the following advances. First, established expert guidelines for the diagnosis and treatment of ATL provide evidence-based recommendations for treatment (Cook et al., 2019). Second, aggressive chemotherapy regimens have been shown to be superior to standard CHOP regimens for treatment of the lymphoma subtype (Tsukasaki et al., 2007). Third, zidovudine plus interferon with or without arsenic trioxide has shown strong responses in the acute subtype of ATL (Bazarbachi et al., 2010). Fourth, allogeneic stem cell transplant has demonstrated potential for cure in eligible subjects (Hishizawa et al., 2010). Fifth, mogamulizumab has shown significant activity in multiple ATL subtypes (Ishida et al., 2015). Sixth, lenalidomide has also shown activity in relapsed and refractory subtypes of ATL (Ishida et al., 2016).

Despite, these advances, the prognosis of ATL remains quite poor. Advances in treatment require the establishment of biomarkers for response predictions. Moreover, improved preclinical models are required for identifying potentially active therapies. The focus of this review is to assess the current status of ATL biomarkers and preclinical models for improving ATL therapy.

## Biomarkers for ATL

Cancer biomarkers refer to a substance or process that is indicative of the presence of cancer in the body (Goossens et al., 2015). A biomarker may be a molecule secreted by a cancer, or a specific response of the body to the presence of the tumor. Genetic, epigenetic, proteomic, and imaging biomarkers can be used for cancer diagnosis, prognosis, or treatment predictions. For the purpose of the current review, we are focused on biomarkers that may provide a quantifiable and accurate predictive measure of ATL response or lack of response to therapy. Ideally, the same biomarker can be used in preclinical and clinical studies. If biomarkers can be identified that are highly predictive of changes in tumor load in patients as well as patient survival, they can assist in defining the mechanism of action of a therapy and/or validating that the intended target is inhibited. In addition, the identification of predictive biomarkers can reduce the cost and duration of future preclinical and clinical studies, and improve safety by decreasing the duration of exposure to ineffective and potentially toxic therapies.

Major prognostic indicators for ATL were defined in a study of 854 patients with ATL (Tsukasaki et al., 2009). The parameters identified by multivariate analysis to have prognostic potential were poor performance status, high lactic dehydrogenase (LDH) levels, age greater than 40 years, more than three involved lesions, and hypercalcemia. Using these factors, a risk model was constructed that may have applications for individual patients. Additional factors associated with a poor prognosis include thrombocytopenia, eosinophilia, bone marrow involvement, high interleukin 5 (IL5) levels in the serum, CC-chemokine receptor 4 (CCR4) expression, lung-resistance-related protein, p53 mutation, and p16 deletion (Tsukasaki et al., 2009). Several markers have also been used to define poor risk chronic ATL (Katsuya et al., 2017).

The proviral DNA level (PVL) may provide prognostic and/or predictive information (Iwanaga et al., 2010). Quantitation of proviral levels in peripheral blood mononuclear cells is performed by quantitative PCR methods, by comparison with a cellular gene (Brunetto et al., 2014). The presence of a single provirus per infected cell allows one to use PVL as a measure of the number of infected cells (Cook et al., 2014). Levels of PVL have been used to follow patients after allogeneic hematopoietic stem cell transplants for ATL (Shiratori et al., 2008). However, it must be recognized that PVL measures the number of infected cells rather than number of malignant cells. Most assays have utilized the *tax* gene for PCR amplification. Moreover, the number of proviruses per peripheral blood mononuclear cell (PBMC) may be higher in acutely infected cells as compared to chronically infected cells. It must also be kept in mind that PBMCs include B lymphocytes, and CD8+ lymphocytes in addition to CD4+ lymphocytes. Although CD4+ lymphocytes are the predominant infected cell *in vivo*, significant viral loads can be detected in CD8+ lymphocytes, which are rarely transformed by HTLV-1 (Goon et al., 2002). In addition, although the primers for PCR are typically based on highly conserved HTLV-1 sequences, polymorphisms, mutations, or deletions within *tax* would affect PVL measurements. Lastly use of a cellular gene as a control may be problematic given high levels of aneuploidy associated with ATL, which may affect the copy number of the chosen cellular gene (Kamihira et al., 1994).

Cell-free circulating tumor DNA (cfDNA) has been used to monitor tumor load in a number of types of malignancies (Corcoran and Chabner, 2018). The precise mechanism of release of cfDNA is unclear. Biological processes suggested to be involved include apoptosis and necrosis from dying cells, or active release from viable tumor cells. The typical size of fragmented cfDNA is 166 base pairs, which corresponds to the length of DNA wrapped around a nucleosome plus a linker (Cole et al., 2016). Various techniques have been proposed for collection, extraction, and analysis of cfDNA (Stewart and Tsui, 2018). A recent abstract presentation described the application of this methodology for monitoring HTLV-1 infection (Haddow et al., 2019). In this study, plasma DNA was isolated as well as genomic DNA from PBMCs from 36 subjects. HTLV-1 DNA was detectable by qPCR in 30 of 36 samples with 0–31 copies of tax/ml plasma (P-cfDNAPVL). A strong correlation was found between P-cfDNAPVL and PBMC-PVL. It should be noted, however, that none of the individuals participating in this study had ATL.

Cell-associated HTLV-1 RNA may also be used to quantify virus gene expression (Yamano et al., 2002). Since helix basic zipper gene (HBZ) is continuously expressed in HTLV-1 infected individuals, it is predicted that levels of *hbz* mRNA may correlate well with virus load (Mitobe et al., 2015). Since *tax* is generally not expressed or only transiently expressed in ATL, detection of *tax* RNA in PBMCs may provide a measure of plus strand expression (Kataoka et al., 2015). Levels of *tax* RNA in PBMCs may be elevated in acutely infected cells, or in cells manifesting virus re-activation from latency. Another measure of active virus replication is the level of 1- or 2-LTR circles (Sloan and Wainberg, 2011). These are considered by-products of reverse transcription and entry of preintegration complexes in the nucleus. The 1- or 2-LTR circles are thought to form as a result of recombination and/or ligation in the nucleus, but are not likely to be templates for integration (Bukrinsky et al., 1993). In one study of HTLV-1 LTR circles, 1-LTR circles were detected in 14 of 20 patients at a mean of 1.38 copies/100 PBMCs. The 2-LTR circles were detected in 30 of 31 patients (Fox et al., 2016).

HTLV-1 infected cells may increase in an individual through (1) retrovirus replication, virus release, and infection of new target cells, and/or (2) clonal expansion of infected cells carrying an integrated provirus that undergo mitotic cell division (Bangham and Ratner, 2015). Although HTLV-1 integrates at a wide range of different sites in the human genome, with limited preference for specific genetic features, ATL cells within an individual are in 91% of cases, characterized by a predominant single integration site (Cook et al., 2014). In 24% of ATL cases an intermediate abundance clone is present. This was first identified by Southern blot analysis of HTLV-1 provirus integration, and more recently by next-generation sequencing methods of integration sites (Yamaguchi et al., 1984; Gillet et al., 2011). Alternatively, variant allele frequency measurements of specific ATL genetic abnormalities provide an alternative measurement of clonal outgrowth of ATL (Farmanbar et al., 2018). Laydon et al. (2014) and Farmanbar et al. (2019) also reported quantification of HTLV-1 clonality using TCR diversity analysis. However, we have identified subjects in whom two or more TCR gene rearrangements are present within a population of ATL cells with a single proviral integration site (Rauch et al., 2019). Moreover, intratumor heterogeneity has been shown to be a complex and early event in the development of ATL (Farmanbar et al., 2018).

Cell surface marker analysis may also be used to monitor tumor load. Levels of survivin, a member of the inhibitor of apoptosis (IAP) family was reported to be predictive of clinical response to proteasome inhibitor bortezomib or anti-CD25 antibody, daclizumab (Pise-Masison et al., 2009). More recently, a multicolor flow method has been described as a useful technique to monitor ATL cells in infected patients. For this purpose, Kobayashi et al., showed that CD4 cells expressing cell adhesion molecule-1 (CADM1), but with reduced expression of cell surface co-stimulatory protein, CD7 represent transformed cells. More recently, CD45RA+ T memory stem (T_SCM_) cells were identified as cancer initiating cell capable of reconstituting ATL clones (Nagai et al., 2015).

Several serum markers have also been used as prognostic or predictive markers. The α-chain of the interleukin 2 receptor (IL-2R; CD25) is over-expressed on ATL cells, and sIL-2R have been used to monitor disease, particularly in Japanese patients (Katsuya et al., 2012, 2017). The soluble form of IL-2R is released as a result of proteolytic shedding from activated T cells. Levels of sIL-2R was reported to be a poor prognostic marker in a wide range of B cell lymphomas (Kusano et al., 2017). CD30 is a member of the tumor necrosis factor (TNF) receptor family that is expressed on activated, but not resting T and B cells, and is associated with signal transduction that leads to activation of nuclear factor kappa B (NFκB). Elevated levels of soluble CD30 are also linked with various T-cell neoplasms, including ATL (Nishioka et al., 2005). OX-40 is another member of the TNF receptor superfamily, that co-stimulates activated T cells following interaction with its own ligand. High levels of OX40 have also been reported as a marker of ATL (Tanaka et al., 2019). Lastly, plasma-associated exosomes may also be useful for monitoring HTLV-1 infection (Jeannin et al., 2018).

In summary, numerous potential biomarkers for ATL have been proposed, but systematic comparison and analysis of their utility in monitoring treatment response remains to be performed.

## Cell Culture Models With ATL Cells

Numerous studies have used HTLV-1 infected cell lines for preclinical evaluation of potential anti-ATL therapies. However, it is unclear whether these cell lines recapitulate the key oncogenic features of ATL. HTLV-1 infected cell lines have been subdivided into Tax-expressing (Tax+) and Tax non-expressing (Tax−) cell lines. It is generally believed that Tax expression is lost in ATL (Kataoka et al., 2015). This has been attributed to escape from immunosurveillance (Hanon et al., 2000). Tax is an important target of CTL responses to ATL. Moreover, Tax expression is associated with expression of other plus strand HTLV genes, which also contribute to ATL immune responses (Bangham and Osame, 2005). Alternatively, Tax has been implicated in the induction of cellular senescence through NFκB hyper-activation (Zhi et al., 2011). Repression of Tax has been suggested as a means of overcoming this inhibitory activity.

Molecular mechanisms that account for loss of Tax expression in 50% of ATL cases include deletions in the integrated provirus in ATL cells, DNA methylation, and/or chromatin modifications associated with epigenomic shut-off of the HTLV-1 genome (Takeda et al., 2004; Taniguchi et al., 2005). It has been suggested that tumors capable of expressing Tax compared to those unable to express Tax, may represent two biologically distinct subtypes of ATL, perhaps analogous to the different latency programs in EBV-associated malignancies (Mahgoub et al., 2018).

Tax has been suggested to be an ATL initiation factor, but it is unclear whether it is required for ATL maintenance (Baratella et al., 2017). It is possible that HBZ is a tumor maintenance factor. HBZ has weak tumorigenic activity, and is continuously expressed in HTLV-1 infected cells in patients (Mitobe et al., 2015; Esser et al., 2017).

Transient expression of Tax from a minority of cells in culture was shown to occur in certain ATL cell lines and primary T cell cultures from HTLV-1 infected patients (Mahgoub et al., 2018). In the MT1 cell line, Mahagoub et al., showed that 0.05–3.0% of cells with bursts of Tax expression at any time, lasting on average 19 hrs. During Tax expression, antiapoptotic and NFκB-related genes were expressed. Moreover, they showed upregulation of Tax expression by cytotoxic stress, such as oxidative stress. Repression of Tax expression with siRNA resulted in apoptosis of the culture. Billman et al. (2017) showed similar bursts of Tax expression in T cell clones from HTLV-1 infected subjects. Using single molecule FISH, they found that 1-10% of cells expressed Tax. Moreover, cells expressing Tax did not express HBZ. In addition, to cellular stress, they found that hypoxia or glucose metabolism also enhanced Tax expression.

Based on these observations, it is recommended that preclinical studies demonstrate activity in ATL cell lines which are Tax-independent, as well as cell lines that are Tax-dependent with transient bursts of Tax. However, it should be noted that improved characterization of ATL cell lines is required. Do these cell lines fully recapitulate the biological and molecular characteristics of ATL in the patients from which they were derived? What cultures conditions provide the closest representation of ATL? Are these characteristics representative of all ATL disease subtypes? Are there specific characteristics that differentiate ATL cells derived from smoldering or chronic subtypes of ATL from acute or lymphoma subtypes? Can these ATL cell lines recapitulate key features of the disease when inoculated into an animal model?

## Mouse Models of ATL

Although rats, rabbits, and monkeys have been used in a small number of preclinical models for ATL, the majority of studies have utilized various mouse models (Lairmore et al., 2005). However, HTLV-1 does not infect or replicate in murine cells. Blocks are present at the level of viral entry and Tax expression, and perhaps other steps in virus replication (Trejo and Ratner, 2000). Thus, an ideal animal model for preclinical studies of HTLV-associated tumors is still lacking. This is particularly important in studies of immune therapies, where distinct features of the microenvironment play a critical role in efficacy.

Genetically engineered mouse (GEM) models provide valuable tools for cancer research (Richmond and Zu, 2008). An advantage of such models is that they allow analysis of many genetic backgrounds using a variety of mouse strains. In addition, the tumor exists in the presence of a competent immune system. These models allow definition of mutations that mimic those identified in human tumors. In addition, they allow one to follow tumor development from early time points. Disadvantages include the fact that a limited number of genes may not reflect the complex heterogeneity of human tumor cells, they are costly and time consuming, and tumor development is often slow and variable.

Several different GEM models have been developed with Tax and/or HBZ, resulting in lymphoproliferative disease (Zimmerman et al., 2011). Ratner et al., expressed Tax with the human granzyme promoter (Grossman et al., 1995). These mice have been engineered with a marker to monitor Tax level non-invasively, which led to observations of dynamic changes in Tax levels over time and induction with various inflammatory stimuli (Rauch et al., 2009a, b). Greene et al., reported on an inducible Tax murine model using EmuSR alpha promoter and a doxycycline-repressible enhancer (Kwon et al., 2005). These mice developed polyclonal lymphocytic inflammation in the skin. Hasegawa et al. (2006) expressed Tax in developing thymocytes using the Lck proximal promoter. After 10 months, the majority of these mice developed hepatomegaly and mesenteric tumors, characterized as diffuse large-cell lymphomas. All of these models demonstrated dependence on NFκB activation (Portis et al., 2001; Kwon et al., 2005; Hasegawa et al., 2006). Injection of the Tax transgenic cells from Hasegawa’s transgenic mice into SCID mice resulted in an aggressive leukemia characterized by “flower” cells, as well as extensive lymph node and skin involvement, similar to the transgenic mice. Cancer initiating cells isolated from these tumors reproduced the disease phenotype (Yamazaki et al., 2009). Treatment of transgenic mice with arsenic and interferon, resulted in Tax protein degradation, and depletion of cancer initiating cells (ElHajj et al., 2010). Bellon et al. (2016) used xenografts from these Tax transgenic tumors to study the anti-proliferative activity of Pim1 kinases inhibitors. ElHajj et al. (2014) showed that synthetic retinoids inhibited Tax transgenic tumor cell growth and induced apoptosis in NSG mice. They ascribed this effect to enhanced degradation of Tax. HBZ transgenic mice also develop lymphoid malignancies, but with a longer latency than Tax transgenic animals, and with an incomplete penetrance (Satou et al., 2011; Esser et al., 2017).

Xenografts of human tumor cell lines in mice (CDX models) have provided valuable models for screening and evaluation of candidate anticancer drugs (Richmond and Zu, 2008). Such models can predict the drug response of a tumor in human patients, and are rapid. The major disadvantage is that the mice are immunocompromised, thus, obscuring the effect of the microenvironment. The severe combined immunodeficiency (SCID) mouse was developed in the 1980s, and has been commonly used for xenograft studies of cancer. Although the non-sense mutation in the protein kinase DNA-activated catalytic polypeptide (Pkrdc) results in loss of B and T lymphocytes in these mice, they retain normal macrophage, dendritic, and natural killer cell functions (Zimmerman et al., 2011). Several different ATL cell lines have been engrafted successfully into SCID mice, including Hut 102, RV-ATL, and SLB-1 cells. Further refinements of the mouse models have incorporated into SCID mice, various defects of innate immune function. These include SCID/beige and NOD/SCID mice, with or without mutations of IL-2Rγ. Ishitsuka et al. (2012) used SCID mice inoculated subcutaneously with Hut 102 cells to assess the activity of the pro-apoptosic drug ABT-737. Maeda et al. (2010) used non-obese diabetic (NOD)/SCID mice injected with Hut 102 cells, to assess growth inhibitory activity of anti-CD30 monoclonal antibodies. Ishikawa et al. (2018) used SCID mice inoculated with Hut 102 cells to demonstrate the effects of inhibitors of mTOR and/or PI3K. Tsumuraya et al. (2011) used SCID mice inoculated with Hut 102 cells to demonstrate inhibition of hippuristanol, a polyoxygenated steroid. They ascribed this effect to inhibition of eukaryotic initiation factor eIF4A. Other studies with Hut102 cells in SCID mice have examined NFκB inhibitors curcurmin, dehydroxymethyl epoxyquinomicin (HDMEQ), and synthetic retinoid NIK-333, mevalonate pathway inhibitor incadronate, combined NFκB and AP-1 inhibitors carotenoid fucoxanthinol, modified galectin hG9NC, and retinoic acid receptor inhibitor tamibarotene (Zimmerman et al., 2011). Additional studies have used MT1, MT2, and TL-Om1 cells to study effects of DHMEQ, and MET-1 cells to examine the effects of IL-2R monoclonal antibody, anti-CD52 antibody campath-1H, anti-CD2 antibody MEDI-507, and flavoperidol (Zimmerman et al., 2011). More recently, Zhang et al. (2015) used EC40515(+) cells to study the activity of JAK inhibitors on subcutaneous growth in NOD/SCID, IL2Rγnull (NSG) mice.

Patient-derived xenograft (PDX) models provide a powerful tool to investigate tumor biology and preclinical activity of potential therapies (Jung et al., 2018). PDX models have the advantage over CDX models in providing cancer-associated stromal cells with primary tumors which may provide a more realistic heterogeneity of tumor cells (Richmond and Zu, 2008). Their major shortcoming is that they are expensive and technically complicated. Kondo et al. (1993) demonstrated successful xenotransplantation into SCID mice of PBMCs from six of eight ATL subjects. Similar models have been used by other investigators subsequently (Phillips et al., 2000; Kawano et al., 2005). Nakamura et al. (2015) reported on the use of NOD/SCID mice null for Jak3 injected with primary PBMCs from ATL patients. The malignant cells were serially transplanted between animals. They used this model to demonstrate activity of the anti-oxidant, pyrrolidine dithiocarbamate (PDTC). In recent work, Xiang et al. (2019) described the development of ATL in mice injected with purified CD4+ cells from patients with ATL in NSG mice, but not with total PBMCs. The latter finding suggests that non-CD4+ cells antagonize ATL development in this model.

NSG mice have been humanized with hematopoietic stem cells isolated from umbilical cord PBMCs based on their expression of CD34 or CD133 (Villaudy et al., 2011; Ikebe et al., 2013; Tezuka et al., 2014). These mice can then be infected by inoculation of irradiated HTLV-1 producer cells. After 6–12 weeks, these animals develop an aggressive lymphoproliferative disorder with many of the manifestations of human ATL, including flower cells, visceral infiltration of CD4+ cells, lytic bone lesions, hypercalcemia, and oligoclonal expansion of infected cells (Villaudy et al., 2011; Tezuka et al., 2014; Xiang et al., 2019). These models have the advantage that they can examine effects of drugs on HTLV-1 replication *in vivo*. However, these models have the disadvantage of long duration and variable levels of humanization, incomplete disease penetrance, and variable levels of virus replication.

## Conclusion

Further studies of biomarkers, cell culture and animal models of ATL are very important to develop improve therapies for this devastating malignancy. Critical analysis of each assay and model in preclinical vs. clinical studies of new therapeutic approaches is warranted. Strengths and weaknesses of each approach should be analyzed, compared, and validated.

## Author Contributions

LR reviewed the literature and wrote the manuscript.

## Conflict of Interest Statement

The author declares that the research was conducted in the absence of any commercial or financial relationships that could be construed as a potential conflict of interest.
